# Clinical Information Systems 2025: Integrating AI, Real-Time Data, and Patient-Centered Care

**DOI:** 10.1055/s-0046-1816611

**Published:** 2026-11-03

**Authors:** Werner O. Hackl, Sabrina B. Neururer, Bernhard Pfeifer

**Affiliations:** 1 Division for Digital Health and Telemedicine, UMIT TIROL - Private University for Health Sciences and Health TechnologyHall in TirolAustria; 2 Department of Clinical Epidemiology, Tyrolean Federal Institute for Integrated Care, Tirol Kliniken GmbHInnsbruckAustria

**Keywords:** Medical Informatics, International Medical Informatics Association, Yearbook, Clinical Information Systems, Artificial Intelligence, Data Science, Telemedicine

## Abstract

**Background:**

The Clinical Information Systems (CIS) section of the IMIA Yearbook of Medical Informatics annually identifies and analyzes high-impact research that shapes the evolution of digital health.

**Objective:**

This synopsis highlights key research trends in CIS, presents best papers from 2024, and reflects on emerging themes including artificial intelligence (AI) integration, interoperability, real-time data use, and telemedicine.

**Methods:**

We performed a systematic literature search across PubMed and Web of Science using a consistent query refined over the past two years. A blinded multi-stage review process, using Rayyan, was applied to screen and select papers. Additional analyses included keyword mapping and co-occurrence networks to visualize research clusters.

**Results:**

Among 5,459 publications retrieved, 15 candidate papers and 3 best papers were selected. Notable themes include the integration of AI in clinical workflows, the maturation of telemedicine, advances in interoperability standards (e.g., HL7 FHIR), and the application of real-time data from wearables and Internet of Things (IoT). Papers also addressed the organizational and regulatory challenges of deploying AI-driven tools and secondary use of health data.

**Conclusions:**

The CIS field continues its transformation into patient-centered, AI-enabled, and interoperable ecosystems. This review highlights the importance of balancing technological innovation with human-centered design, governance, and regulatory frameworks. Digital maturity remains a key driver of improved clinical outcomes and safety. Future CIS development should emphasize seamless data integration, secondary data use, and real-time analytics to optimize patient care and system efficiency.

## 1. Introduction

As editors of the Clinical Information Systems (CIS) section, we continue to apply a standardized, systematic search and review process each year to identify outstanding contributions in the field. For the 2025 edition, we once again performed a comprehensive literature search across PubMed and Web of Science, adhering to the refined query strategy introduced in recent years. This query – which explicitly includes telemedicine-related terms and excludes irrelevant topics – remained unchanged from last year, ensuring consistency in our approach. Using this stable search strategy and a multi-stage selection process, we scanned the 2024 literature for novel insights and ultimately selected the best papers for inclusion in the IMIA Yearbook of Medical Informatics. By maintaining the same query and review methodology as in previous years, we aimed to produce a reliable snapshot of current trends and advances shaping the CIS domain.

Our review of the past year’s publications highlights several continuing trends in the evolution of CIS. Foremost, the integration of artificial intelligence (AI) and data science techniques into CIS remains on an upward trajectory. Building on momentum from prior years, we observe an especially growing role for natural language processing (NLP) and the emergence of large language models (LLMs) in the clinical context. These technologies are increasingly being used to extract actionable insights from unstructured clinical data, assist in documentation, and enhance decision support at the point of care. The prominence of AI-driven analytics and advanced NLP applications in this year’s CIS literature underscores a broader push to leverage big data and machine learning to improve clinical workflows, prediction, and personalization in healthcare.

In parallel, we are witnessing a broader transformation of CIS from static documentation systems into intelligent, patient-centered platforms that enable precision care. Whereas legacy electronic health records (EHRs) were often seen as digital filing cabinets for patient information, modern CIS are evolving into proactive tools that generate knowledge and support informed decision-making tailored to individual patients. The patient has truly taken center stage in these systems: personal health data are continuously aggregated and analyzed to create value for the patient’s own care. As a result, today’s CIS serve as the foundation of complex, trans-institutional information logistics processes, connecting data across care settings and delivering context-rich, personalized insights at the bedside. This shift from mere data capture to active data utilization exemplifies how CIS are becoming intelligent platforms that not only document care, but also drive improvements in care quality and outcomes through precision, timeliness, and patient-centric design.

Several enduring factors continue to shape the evolution of CIS. Achieving seamless interoperability remains a paramount goal: hospitals and health systems are working to better exchange information across disparate platforms and institutions, enabling a more holistic and unified view of patient health. There is also a heightened emphasis on real-time data usage within CIS. Whether through integration of streaming vital signs from bedside monitors or incorporation of patient-generated data from wearable devices and remote sensors, CIS are increasingly handling real-time data to support timely clinical interventions. Additionally, the secondary use of health data has become more prominent. Data originally collected in the course of care are now frequently repurposed for research, quality improvement, public health surveillance, and the development of AI models. These drivers – interoperability, real-time data integration, and expanded secondary data use – are collectively pushing CIS to be more connected, responsive, and versatile, further solidifying their role as critical infrastructure for both patient care and health data science.

Finally, it is important to situate this year’s CIS synopsis in the context of the 2025 IMIA Yearbook special topic: *“Digital Health and Climate Change”* . Each year, the IMIA Yearbook highlights a special theme in medical informatics, and section editors consider how this theme manifests in their domain. Accordingly, we examined whether any intersections between digital health and climate change emerged in the CIS-related literature of the past year. This reflective lens prompted us to consider, for example, how telemedicine and remote care might contribute to sustainability by reducing patient travel, or how large-scale health data could be used to track climate-related health impacts. While climate change is not traditionally a core focus within CIS, the growing awareness of environmental factors in healthcare led us to remain attentive to any relevant threads in this year’s selected papers. In the following sections of this synopsis, we present the results of our literature review and highlight the significant papers and developments that exemplify the state of the art in CIS, against this backdrop of ongoing digital transformation and emerging global challenges.

## 2. About the Paper Selection

The selection process for relevant publications in the CIS section follows a rigorous and well-established systematic approach. This year, we employed the same refined search query from 2023 and 2024 [ 1 , 2 ], conducting the search in mid-January 2025. Our search retrieved a total of 5,459 unique articles—4,878 from PubMed (comprising 2,140 from the legacy query and 2,738 from telemedicine-related search terms), and 581 additional articles from Web of Science® (WoS). These articles spanned almost 1,500 journals, with the top 20 journals by publication volume listed in Table 1 .

**Table 1:** Number of retrieved articles for top-20 ranked journals.

**Rank**	**Journal**	**Number of papers**
1	Telemedicine Journal and e-Health: The Official Journal of the American Telemedicine Association	265
2 3	Journal of Medical Internet Research	203
Journal of Telemedicine and Telecare	170
4	Expert Opinion on Drug Safety	156
5	PLOS One	78
6	Digital Health	77
7	JMIR Formative Research	69
8	Health Communication	67
9	Scientific Reports	60
10	BMC Medical Informatics and Decision Making	59
11	BMC Health Services Research	57
12	JMIR mHealth and uHealth	56
13	International Journal of Medical Informatics	55
14	Journal of the American Medical Informatics Association: JAMIA	47
15	JMIR Human Factors	46
16	BMJ Open	45
17	JAMA Network Open	43
18	Journal of General Internal Medicine	41
19	Applied Clinical Informatics	35
20	JMIR Medical Informatics	31

The distribution of countries of origin for the publications was similar to previous years, with the United States and England again accounting for the majority of the contributions. Specifically, the United States contributed 41% (n=2,002) of the publications, followed by England with 33% (n=1,579). Canada ranked third with 6% (n=312), while the Netherlands, Germany, and Switzerland each contributed 3% (n=167; 166; 150) of the papers. Ireland also featured prominently, contributing 2% (n=106). Australia again rounded out the group of countries with more than 50 publications, contributing 2% (n=92).

As in previous years, we used the online systematic review tool RAYYAN 1 for the blinded, multi-stage selection process of the best papers. Each section editor independently and blindly reviewed all papers, applying an initial exclusion step based on titles, keywords, journals, and abstracts—narrowing the pool to 211 articles. Papers were then further reviewed through mutual consent and, in several cases, additional full-text reading, leading to the identification of 25 possible candidates. From this, 15 plus one additional candidate were shortlisted. The final selection of the best papers was made during the editorial board meeting on May 7, 2025, in Paris, France. The best papers are displayed in Table 2 and their summaries are provided in the appendix of this synopsis.

**Table 2:** Best paper selection of articles for the IMIA Yearbook of Medical Informatics 2025 in the CIS section. The articles are listed in alphabetical order of the first author’s surname.

Section : Clinical Information Systems
Kruse J., Wiedekopf J., Kock-Schoppenhauer A.-K., Essenwanger A., Ingenerf J.,Ulrich, H. (2024). A Generic Transformation Approach for Complex Laboratory Data Using the Fast Healthcare Interoperability Resources Mapping Language: Method Development and Implementation. JMIR Medical Informatics, 12, e57569. https://doi.org/10.2196/57569 . Priou S., Kempf E., Jankovic M., Lamé, G. (2024). “Goldmine” or “big mess”? An interview study on the challenges of designing, operating, and ensuring the durability of Clinical Data Warehouses in France and Belgium. Journal of the American Medical Informatics Association : JAMIA, 31(11), 2699–2707. https://doi.org/10.1093/JAMIA/OCAE244 Snowdon A., Hussein A., Danforth M., Wright A., Oakes, R. (2024). Digital Maturity as a Predictor of Quality and Safety Outcomes in US Hospitals: Cross-Sectional Observational Study. Journal of Medical Internet Research, 26, e56316. https://doi.org/10.2196/56316

## 3. Findings and Trends: Clinical Information Systems and Telemedicine Research 2024

As in previous years, we used text mining and bibliometric network visualizations to map relationships and trends among publications. This year, we again applied these techniques to create a clear and concise overview of the selected papers and key insights.

### 3.1. Overview of the Content of all Found CIS Papers

As in previous years, we created a tag cloud from the authors’ keywords to gain an initial overview of the content of the publications—a method that has consistently proven valuable. This year, we again extracted 43,105 keywords from all articles and generated a tag cloud using the top 250 of them ( Figure 1 ).

**Figure 1.**
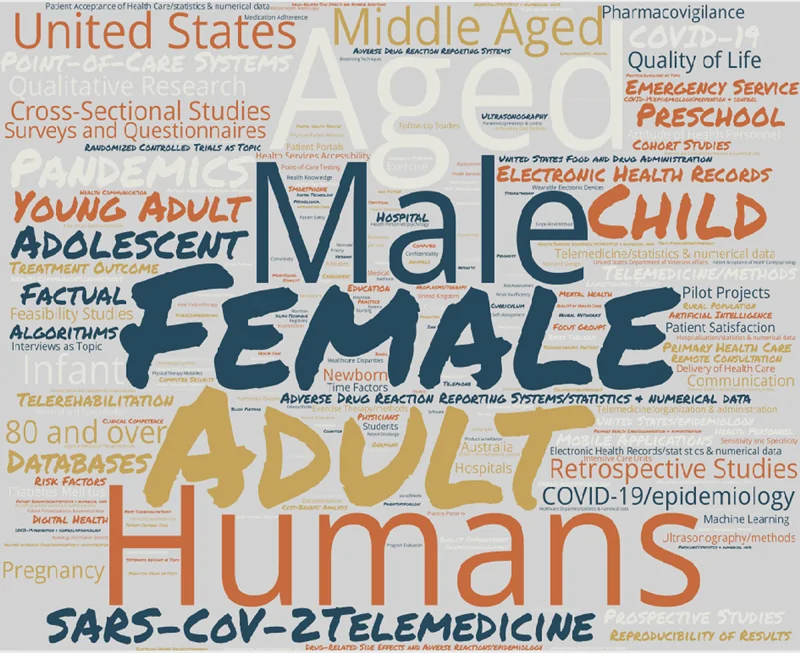
Tag cloud illustrating the frequency authors’ keywords (top 250 keywords out of n=43,105 are shown) within the 5,459 papers from the CIS query result set. Font size corresponds to frequency (the most frequent keyword was „humans“, n=3,977).

As in previous years, we also created a bibliometric network alongside the tag cloud to visualize the connections and interrelations between the publications. We once again relied on the well-established tool VOSviewer [ 3 ] and applied comparable settings as last year to generate a clustered co-occurrence map of the keywords, which is shown in Figure 2 .

**Figure 2.**
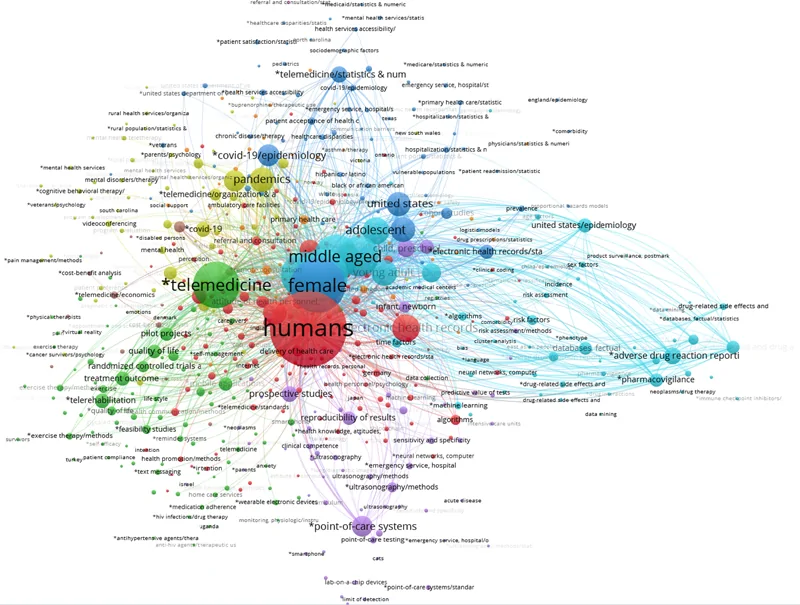
Clustered co-occurrence map of the top-562 keywords (keywords with the greatest total link strength, n=562 of 6,303 distinct keywords) from the 5,459 papers in the CIS query result set. Only keywords that we found in at least seven different papers were included in the analysis. Node size corresponds to the frequency of the keywords (for “humans”, n=3,977). Edges indicate co-occurrence (only the top 1,000 of 18,122 edges are shown). The distance of nodes corresponds to the link strength between the keywords. Colors represent the nine different clusters. The network was created with VOSviewer [ 3 ].

The resulting map is very similar to last year’s, featuring nine distinct clusters that capture the various perspectives and aspects of the studies. To ensure consistency with previous years, we also conducted a parallel analysis using terms extracted from the titles and abstracts of all publications. The resulting co-occurrence map of the top 507 terms is shown in Figure 3 .

**Figure 3.**
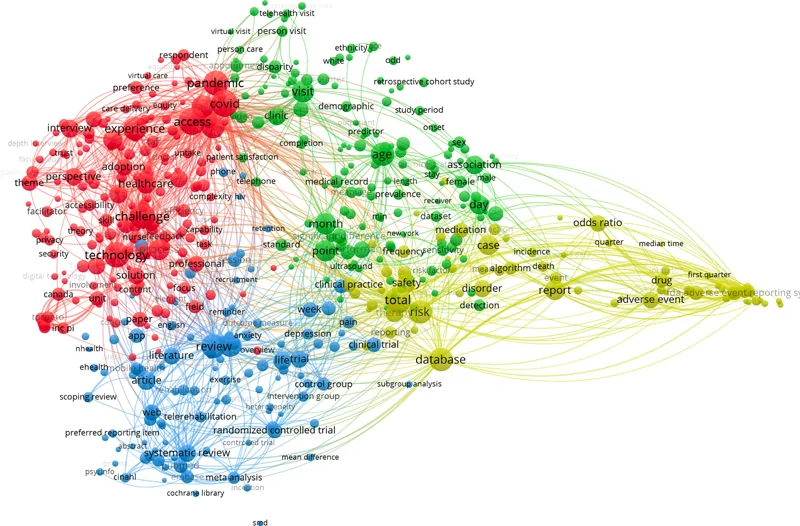
Clustered co-occurrence map of the top-500 terms (top 60% of the most relevant terms: n=507 of 105,180) from the titles and abstracts of the 5,459 papers in the CIS query result set. Only terms that we found in at least 38 different papers were included in the analysis. Node size corresponds to the frequency of the terms (binary count, once per paper, “access”: n=949, “covid”: n=944). Edges indicate co-occurrence (only the top 1,000 of 94,892 edges are shown). The distance of nodes corresponds to the association strength of the terms within the texts. Colors represent the four different clusters. The network was created with VOSviewer [ 3 ].

This year, the overall picture is once again largely unchanged from previous years, with clusters displaying familiar structures and thematic elements. Consequently, there is little additional insight to be gained from this high-level view. Therefore, we proceed with a detailed examination of the central publications selected for this year’s CIS section, which are presented in the next sections.

### 3.2. Insights into the Candidate Papers and Best Papers

The following summaries highlight the most significant contributions and share the key insights gained from our analysis of the current research landscape in CIS.

#### 3.2.1. AI in Clinical Decision Support

AI integration into clinical workflows and education was a prominent theme this year. Zhui et al. explored the ethical challenges of deploying large language models (LLMs) in medical training, such as AI hallucinations, bias, privacy risks, and lack of transparency [ 4 ]. The authors emphasize the need for ethical frameworks and principles to guide LLM use (e.g., protecting patient privacy despite LLMs’ tendency to memorize sensitive data), reflecting the CIS trend of AI and LLM integration with a focus on responsible design. Baxter et al. evaluated the use of GPT-based LLMs to draft replies to negative patient messages in an EHR [ 5 ]. Their initial findings showed that AI-generated drafts are feasible as a starting point to reduce clinician workload, but often lacked a personal clinical tone and could occasionally escalate emotional conversations inappropriately. For example, some ChatGPT drafts were overly generic or even suggested the patient lodge a complaint, indicating the need for human oversight and refinement of AI replies. This work highlights both the promise of AI in streamlining communication and the patient-centered challenges (tone, empathy) that must be addressed.

Lampreia et al. reviewed the use of digital health technologies (wearables, mobile apps, telemedicine) and AI in European cardiovascular trials [ 6 ]. They found a rapid post-pandemic increase in trials leveraging these tools and noted that when such digital interventions serve clinical decision-making (e.g., AI-guided monitoring), they may become regulated as medical devices. The authors underscore the importance of robust validation and regulatory frameworks for AI-driven digital trials to ensure safety and efficacy.

Additionally, Naimark et al. implemented an EHR-based pre-visit questionnaire to help patients articulate their healthcare goals [ 7 ]. Their mixed-methods evaluation demonstrated moderate patient uptake, with real-world challenges in embedding patient goals into clinical workflows. Despite initial barriers (such as patients forgetting to print or bring the summaries), integration directly into the Epic EHR improved completion rates. Clinician feedback emphasized the need for seamless workflow integration and patient support, highlighting ongoing challenges in achieving patient-centered care through digital means.

Niu et al. introduced EHR-BERT, a deep learning model for detecting anomalies in EHRs [ 8 ]. This model demonstrated that advanced NLP techniques can effectively support data quality management in CIS, helping to identify errors and inconsistencies in large-scale health data—a crucial step toward reliable AI-driven decision support. Collectively, these papers demonstrate the expansion of AI applications in decision support – from education to patient-facing tools – while emphasizing ethics, oversight, and validation as key to sustainable integration into clinical systems.

#### 3.2.2. Data Interoperability and Standards

Achieving seamless data exchange and interoperability remains a core CIS focus. Notably, Snowdon et al. showed that advanced digital maturity correlates with automated data flow across systems and better safety outcomes – underscoring how interoperability contributes to quality [ 9 ].

Kruse et al. addressed the technical challenge of standardizing complex lab results [ 10 ] (selected as best paper). They developed FML2Mirth, a tool that uses HL7 FHIR’s mapping language to automatically convert proprietary lab data into standardized FHIR-compatible formats. In practical tests, FML2Mirth successfully generated interoperable lab reports (HL7 and national format) from legacy lab files, demonstrating the feasibility of automating data transformation for interoperability. The tool integrates a terminology server, and validation checks to preserve data fidelity during conversion. Additional studies emphasized the importance of structured data integration for secondary use and cross-institutional research.

Sokol examined the European Health Data Space (EHDS) regulation, advocating for an opt-out mechanism to balance data sharing with patient privacy [ 11 ]. This regulatory perspective reinforces the need to align data exchange initiatives with ethical and legal frameworks—an important consideration in CIS development.

#### 3.2.3. Real-Time Data Use and IoT Integration

Several papers highlighted the use of real-time data from wearables and Internet of Things (IoT) in clinical research and care. Pulantara et al. introduced the ADAM platform, a flexible architecture for integrating data from fitness wearables, smartphone apps, and IoT sensors in behavioral health trials [ 12 ]. ADAM supports real-time data acquisition via device APIs and adaptive mobile questionnaires, and also functions as a trial management system (recruitment, randomization, monitoring). They deployed ADAM in a 500-participant weight-loss randomized controlled trial (the SMARTER trial), which required rapid adaptation to fully remote operations during COVID lockdowns. The system was successfully and efficiently implemented, enabling seamless remote assessments and data tracking without disrupting the trial. In fact, ADAM’s real-time integration allowed the study to continue with minimal impact during pandemic restrictions, highlighting its robustness. The authors report that ADAM’s modular design is scalable and generalizable to other interventions, demonstrating how real-time, interoperable data infrastructures can support large-scale digital health research.

Tang et al. contributed with a comprehensive review of EHR-based research methodologies, emphasizing the challenges of data biases and the importance of robust validation to ensure meaningful integration of real-world data into CIS [ 13 ]. These studies exemplify the Yearbook’s real-time data use trend, showing how continuous data streams can be captured and utilized in clinical studies, supporting just-in-time adaptive therapies and enabling more patient-centered, responsive interventions.

#### 3.2.4. Secondary Data Use and Digital Health Impact

A significant trend in 2024 was leveraging large-scale health data to evaluate the impact of CIS on quality and outcomes. Snowdon et al. [ 9 ], which we also highlighted earlier as one of this year’s best papers, plays an important role here too. Their study linked advanced EHR maturity to significantly better safety results, illustrating how health IT investments translate into improved patient outcomes.

Complementing this, Modi et al. examined the financial benefits of EHR adoption in relation to hospital performance [ 14 ]. They found that financial benefits of EHRs emerged only when hospitals effectively used them to reduce readmissions.

Priou et al. provided a qualitative study on the challenges of designing and operating clinical data warehouses (CDWs) in France and Belgium [ 15 ] (selected as best paper). Their insights revealed that misaligned stakeholder expectations and inadequate knowledge management hamper the long-term sustainability of CDWs—a crucial lesson for advancing secondary data use infrastructures. Together, these papers illustrate the potential of secondary data use to drive improvements in quality, safety, and financial performance, while also highlighting the importance of governance and stakeholder alignment.

#### 3.2.5. Telemedicine and Remote Care

Building on the pandemic-driven expansion of telehealth, multiple papers investigated the sustainability, equity, and efficacy of remote care models. Chandrasekaran analyzed outpatient telemedicine utilization in the immediate post-COVID era [ 16 ]. This work probed whether the surge in telehealth during COVID-19 was maintained and equitable across patient groups. The analysis revealed that telemedicine use did persist after the pandemic’s peak, but utilization was uneven: younger, more affluent, and tech-enabled populations tended to use telehealth more, whereas some disadvantaged groups lagged, indicating persistent digital divide issues. In short, telemedicine’s benefits did not uniformly reach all demographics, highlighting equity gaps that need to be addressed even as virtual care becomes routine.

In contrast, Zychlinski et al. demonstrated the remarkable potential of telemedicine to improve outcomes [ 17 ]. In a study comparing 159 acutely ill patients treated via a telemedicine-supported Hospital-at-Home (HAH) program to matched inpatients, the HAH model outperformed traditional hospitalization on several key metrics. HAH patients had a significantly shorter length of stay and no increase in readmission rates, and for both COVID-19 and non-COVID cohorts the mortality risk was significantly lower with home care. The authors conclude that, when implemented with careful patient selection, 24/7 telemonitoring by experienced physicians, and appropriate technology, HAH can be superior to standard in-hospital care for many patients – a paradigm shift from the initial assumption that it would be merely non-inferior. This pioneering study underscores telemedicine’s capacity to expand acute care beyond hospital walls without compromising safety, aligning with the trend of patient-centered care delivered in the home.

Focusing on the patient experience, Verma et al. evaluated 9,862 post-visit surveys to compare satisfaction between video visits and in-person visits in primary care [ 18 ]. Reassuringly, they found no significant differences in core satisfaction measures: patients rated telehealth visits just as highly as in-person visits in terms of clinician communication, time spent, and likelihood of recommending the practice. In fact, telemedicine scored slightly higher in convenience-related aspects – patients reported better ease of getting an appointment and contacting the office by phone when using telehealth, presumably due to reduced logistical barriers. The authors conclude that virtual visits achieved parity in patient satisfaction with traditional care, reinforcing that telemedicine can maintain care quality from the patient’s perspective.

Additionally, Falis et al. investigated the potential of generative AI in clinical documentation and coding—relevant to remote care documentation challenges. They explored the use of GPT-3.5 to generate synthetic discharge summaries for rare ICD-10 codes, aiming to augment data for training local models [ 19 ]. Their study showed that synthetic summaries modestly improved performance in rare code classification and reduced out-of-family errors, though overall performance on real test sets was slightly lower than models trained only on real data. Importantly, clinicians rated 72% of synthetic summaries as correct and 60% as useful in practice. This highlights the potential of generative AI for augmenting training data in telehealth and remote care documentation, while also underscoring the importance of careful evaluation of synthetic data before deployment in clinical contexts.

Taken together, these telehealth studies highlight that remote care has become an integral component of modern clinical systems. They address critical dimensions: access and equity (ensuring all patient groups can benefit), clinical efficacy (meeting or exceeding outcomes of in-person care), and patient satisfaction. Additionally, the findings hint at important co-benefits aligned with the Yearbook’s 2025 special theme of Digital Health and Climate Change. For instance, telemedicine consistently yields travel reductions for patients, which not only saves time and cost but also lowers carbon emissions from transportation. As healthcare strives to reduce its environmental footprint, the expansion of high-quality virtual care—as evidenced by sustained use post-pandemic, effective HAH models, and the use of generative AI to support remote documentation—could contribute to more climate-friendly healthcare delivery by reducing unnecessary in-person visits and associated travel. This synergy between digital health innovation and sustainability adds a forward-looking perspective to the CIS field, illustrating how the patient-centered and data-driven advances of 2024 can also support global goals in the years ahead.

As in previous years, we would like to draw the readers’ attention to this year’s survey paper from the CIS section. Thomas Schabetsberger, Patrick Mangesius, and Alexander Hörbst have prepared a comprehensive analysis on the role of eHealth and telemedicine applications in climate change mitigation—examining health information exchange, care collaboration, and patient engagement [ 20 ].

## 4. Conclusions and Outlook

This 2025 synopsis of the CIS section of the IMIA Yearbook reflects a dynamic landscape where CIS are evolving from passive data repositories into intelligent, patient-centered platforms. The selected best papers illustrate significant progress in key areas: AI integration for clinical decision support, real-time data use from wearables and IoT, and advancements in interoperability frameworks like HL7 FHIR. These developments are paving the way for more personalized and efficient healthcare delivery.

A strong emphasis this year was placed on responsible AI adoption, highlighting the importance of ethical frameworks, transparency, and human oversight to ensure safe and effective integration into clinical practice. Equally, the expansion of real-time data architectures demonstrated the value of continuous monitoring and remote interventions, contributing to both patient-centered care and potential environmental benefits by reducing the need for in-person visits.

Interoperability remains a critical focus, with several papers demonstrating practical solutions to bridge data silos and support secondary use of health data. The increasing role of CIS in facilitating large-scale data integration is essential for supporting research, quality improvement, and cross-institutional collaboration.

Looking forward, the CIS field must continue to prioritize interdisciplinary collaboration, regulatory alignment, and user-centered design to translate technological advancements into real-world clinical benefits. With ongoing attention to ethical, technical, and human factors, CIS will remain a cornerstone of modern healthcare transformation, supporting not only care delivery but also learning health systems and sustainable, climate-friendly care models. The growing attention to sustainability highlights how digital health innovations can also contribute to addressing the challenges posed by climate change.

## Footnotes

1
https://www.rayyan.ai
